# Editorial: World Malaria Day 2023 - ending malaria transmission: reaching the last mile to zero malaria

**DOI:** 10.3389/fpubh.2024.1433213

**Published:** 2024-06-10

**Authors:** Vas Dev, Kinley Wangdi

**Affiliations:** ^1^ICMR – National Institute of Malaria Research, New Delhi, India; ^2^HEAL Global Research Centre, Health Research Institute, Faculty of Health, University of Canberra, Bruce, ACT, Australia; ^3^National Centre for Epidemiology and Population Health, College of Health and Medicine, Australian National University, Acton, ACT, Australia

**Keywords:** public health, malaria control and elimination, malaria parasite, vector resistance, emerging challenges

World Malaria Day is commemorated globally every year on 25 April with the overarching goal of mobilizing all efforts to corner malaria with the aim of eliminating it by 2030. In this context, Frontiers has launched an initiative to coincide with the theme, “Ending malaria transmission: reaching the last mile to zero malaria” and compiled five articles advocating for investment, innovation, and the implementation of newer interventions to defeat malaria.

Driven by the World Health Organization's (WHO) Global Technical Strategy (GTS) for malaria 2016–2030 (1), as many as 43 countries have been certified malaria-free, while several others have achieved marked reductions in morbidity and mortality paving the way for malaria elimination. In the midst of this success story, the WHO reported an estimated 249 million malaria cases in 2022 (an increase of 5 million cases from the preceding year) and 608,000 deaths (2). However, the majority of these cases (94%) and deaths (95%) occurred in the WHO African Region followed by the WHO South-East Asia Region with 2% of cases and 1.31% of deaths respectively. Nearly half of the cases in 2022 were reported by just four countries: Nigeria (27%), the Democratic Republic of the Congo (12%), Uganda (5%) and Mozambique (4%). Of these, 11 countries classified as “high burden to high impact (HBHI)” contributed 67% of estimated cases and 73% of deaths.

Certainly, significant advancements have been made toward malaria elimination, but the last mile of malaria elimination is beset with a host of challenges some of which include: (i) deletion of the histidine-rich protein 2 and 3 (*Pfhrp*2/3) gene (known to exist at high frequencies in many endemic countries), which can result in evasion of detection of *Plasmodium falciparum* (the killer parasite) by rapid diagnostic tests (RDTs), leading to continued transmission (3), (ii) fast evolving resistance to antimalarial drugs in *P. falciparum* malaria (4), (iii) emerging insecticide resistance to multiple insecticides in a host of vector species spread across continents (5), (iv) invasion and spread of *Anopheles stephensi* (urban vector able to endure high temperatures and resistant to a wide array of insecticides) in the African continent (6), (v) climate change as a multiplier of threats resulting in proliferation and spread (e.g., fivefold increase in cases in Pakistan due to devastating floods in 2022 is attributed to climate change) (2), and (vi) emergence of zoonotic malaria *P. knowlesi* (fifth parasite which is known to cause severe and rapid onset of disease in humans) with implications for malaria-free certification (7). It appears like the last mile to defeat malaria is not going to be an easy walk. Instead, it is going to take too long before we achieve the goal of living in a malaria-free world.

Despite all these challenges, several countries have made commendable progress in reducing malaria incidence and mortality post-GTS. In a renewed commitment to defeat malaria, other landmark developments include: (i) the rollout of the first malaria vaccine RTS, S/AS01 (Mosquirix), and the recommendation of a second vaccine R21/Matrix-M; it is hoped that two vaccines on the market will improve access and reach the marginalized and vulnerable (8); (ii) the deployment of a new generation of dual-active pyrethroid-chlorfenapyr insecticide-treated nets (ITNs) to overcome emerging pyrethroid resistance (9); and (iii) the scale-up of seasonal chemoprophylaxis to prevent malaria in those more at risk, resulting in an increasing number of countries advancing toward elimination or achieving certification.

Eliminating malaria is not only desirable but possible, and all options including a subnational approach using situation-specific intervention strategies that move away from the “*one-size-fits-all*” approach should be explored. In this context, Rajvanshi et al. demonstrated the role of “integrated approaches” incorporating current intervention tools, community involvement, and public-private collaborations in achieving the near elimination of indigenous transmission in the malaria-endemic high-risk district of central India making a strong case for implementation in other endemic regions. Furthermore, Singh et al. reported a similar success story and strongly advocated for the intensification of interventions to defeat malaria in high-transmission settings with the goal of elimination by 2030.

Nevertheless, meeting these targets by 2030 appears to be a distant reality. Developing more effective tools will be essential to reaching the last mile in the fight against malaria. Given the disease burden and complex biology of *P. vivax*, Kumar et al. advocated multiple interventions to tackle this resilient parasite, which may prove to be the stumbling block to achieving elimination. However, universal population coverage coupled with appropriate funding and community compliance, is central to ending disease transmission. In this context, Negasa et al. underscored that while long-lasting insecticidal nets are widely recognized as an effective intervention in varied transmission settings, these remain underutilized among vulnerable populations calling for concerted efforts by stakeholders to educate at-risk communities for enhanced compliance.

On the road to malaria elimination, the advent of malaria vaccines (particularly the R21/Matrix-M vaccine, which shows a high level of efficacy) is indeed a welcome development that adds to the arsenal that helps prevent malaria infection; however, there are several issues related to its reach universalization. In this context, Dzi has addressed the subject and strongly advocated for its integration with available intervention tools to realize the common goal of freedom from malaria. In keeping with the Global Malaria Programme Operational Strategy 2024–2030 (10), it is an opportune time to scale up interventions to achieve zero malaria for the benefit of future generations.

## Author contributions

VD: Writing – original draft, Writing – review & editing. KW: Writing – original draft, Writing – review & editing.
